# 47,XXY/48,XXXY/49,XXXXY mosaic with hydrocephaly: a case report and review of the literature

**DOI:** 10.1186/1752-1947-1-94

**Published:** 2007-09-19

**Authors:** Jesús E Dueñas-Arias, Maribel Aguilar-Medina, Eliakym Arámbula-Meraz, Juliana B Valenzuela-Camacho, Angelina Vega-Solano, Julio Granados, Rosalío Ramos-Payán

**Affiliations:** 1 Departamento de Genética, Hospital Pediátrico de Sinaloa. Culiacán, Sinaloa, México; 2 Laboratorio de Inmunología y Biología Molecular, Doctorado en Biotecnología, Facultad de Ciencias Químico Biológicas, Universidad Autónoma de Sinaloa. Culiacán, Sinaloa, México; 3 Departamento de Inmunología y Reumatología, Instituto Nacional de Ciencias Médicas y Nutrición Salvador Zubirán, México D.F., México

## Abstract

Klinefelter's syndrome is a frequent genetic sexual alteration in males, associated with the 47,XXY aneuploidy. Several syndrome variants are caused by different X and Y polysomy and mosaicisms, including the 49,XXXXY condition described by some authors as Fraccaro's syndrome. Mosaics with three or more different chromosomal lines are very rare. Here, we describe a case with XXY/XXXY/XXXXY mosaic in a newborn with clinical features of Fraccaro's syndrome, but also with obstructive hydrocephaly which has not been reported previously.

## Background

Klinefelter's syndrome is a common sex chromosomal abnormality observed in humans, with a prevalence of 1 in 500 males [1-3]. The clinical features are variable but often include infertility, gynecomastia, eunuchoidism, small testes and penis and hypergonadotropic hypogonadism. The syndrome is usually caused by the presence of one additional X chromosome (47,XXY aneuploidy), however, rare syndrome variants with X and Y polysomy, mosaicisms and aberrant chromosomes have been reported, including 46,XX, 48,XXXY, 48,XXYY, 49XXXXY, 47,XXY/48,XXXY and 48,XXXY/49,XXXXY among others [4].

The 49,XXXXY chromosomal constitution was described by Fraccaro in 1960 [5]. Despite being usually considered as a Klinefelter variant, the 49,XXXXY aneuploidy shows a distinct phenotype and more severe clinical features [6,7]. More than one hundred cases have been reported, with a frequency of 1 in 85,000 males [1,8].

Klinefelter with mosaicisms presents a moderate phenotype and accounts for 15% of all cases [6]. The 47,XXY/48,XXXY/49,XXXXY is a very rare mosaic, and to our knowledge, only three cases have been reported until now [9-11]. In this paper, we described XXY/XXXY/XXXXY mosaicism in a newborn with congenital obstructive hydrocephalus and mild cardiopathy.

## Case presentation

Clinical history. A 5-day old male was admitted at the Pediatric Hospital of Sinaloa in México. His parents were unrelated. He was born by cesarean section at week 39 due to fetal arrhythmias and intrauterine growth retardation. He had an Apgar score of 8 at 1 minute and 9 at 5 minutes, 2 Kg birth weight, 47 cm body length and 37 cm cephalic circumference. The patient was hospitalized because clinical examination revealed several congenital abnormalities: hypotrophy, macrocephaly, facial asymmetry, hypertelorism, low nasal bridge, low-set ears, micrognathia, short neck, narrow thorax, bulky abdomen, clinodactyly of the fourth and fifth fingers of both hands, hyperpigmented genitals, micropenis, hypospadia and a mid-scrotal septum with left cryptorchism.

**Figure 1:**
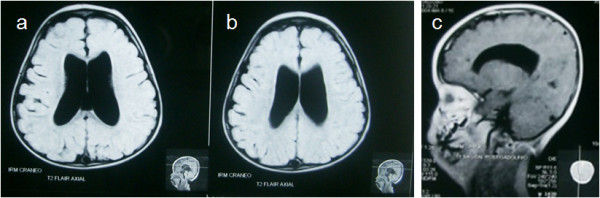
**Magnetic Resonance of head (MR-Head)**. MR-Head showing supratentorial non-communicating hydrocephalus (a, b) and slightly hypoplastic corpus callosum (c).

Laboratory data. Radiographs showed no osseous abnormalities in joints and long bones. Echocardiography revealed a 1.6 mm arterial vessel with bidirectional flux and atrioventricular concordance. Abdominal ultrasound was normal, but anomalies were observed in the transfontanelle ultrasonography. Magnetic resonance imaging of the head confirmed the presence of supratentorial non-communicating hydrocephaly with slightly hypoplastic corpus callosum (figure 1).

**Figure 2:**
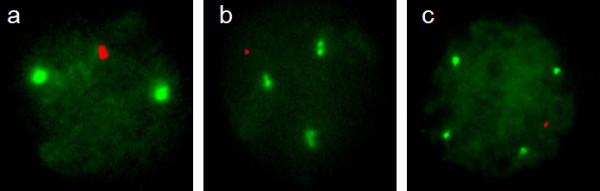
**Fluorescence In situ Hybridization (FISH) assay**. Nuclei FISH analysis of peripheral lymphocytes reveals a XXY/XXXY/XXXXY mosaic in a, b and c respectively.

Genetic analysis. Cytogenetic study by GTG banding of peripheral lymphocytes showed a XXY/XXXY/XXXXY mosaicism (data not shown). FISH analysis of 207 cellular nuclei, using probes for chromosomes X and Y, indicated a line proportion of 3% for 47,XXY, 36% for 48,XXXY and 61% for 49,XXXXY (figure 2).

## Discussion

Fraccaro's syndrome is caused by 49,XXXXY chromosomal aneuploidy and is a rare condition often classified as a Klinefelter's syndrome variant, however, as stated by Peet and Hou, we propose that it should be diagnosed as an independent clinical syndrome [8,7].

In this paper, we describe a newborn with a XXY/XXXY/XXXXY mosaicism with Fraccaro's syndrome phenotype. The percentage of the 49,XXXXY line was 61% as determined by nuclei FISH analysis (figure 1), which is in accordance with the clinical features of the patient. To our knowledge, this is the fourth report of this mosaicism in the literature, but with different line proportion and clinical phenotypes. Moreover, the child had mild cardiopathy and supratentorial non-communicating hydrocephalus (figure 2). Whether this congenital hydrocephaly is a new syndrome variant or just an independent event remains to be determined. As the presence of hydrocephaly in patients with XXY/XXXY/XXXXY mosaicism has not been reported before in any Klinefelter variants nor in Fraccaro's syndrome, it should be considered in future cases, during cytogenetic analysis and in the first six months after birth.

## Competing interests

The author(s) declare that they have no competing interests.

## Authors' contributions

DAJE provided genetic counselling to the parents. AMM and DAJE collected the data relative to this case report. VMJB and VSA conducted the data analysis and interpreted experiments and revised the manuscript. AMM, AME, GJ and RPR performed genetic studies and elaboration and drafting of the manuscript. All authors read and approved the final manuscript.

## Consent

Written consent was obtained from the newborn's mother for publication of this case report.
